# Prediction of gait recovery in spinal cord injured individuals trained with robotic gait orthosis

**DOI:** 10.1186/1743-0003-11-42

**Published:** 2014-03-24

**Authors:** Xun Niu, Deborah Varoqui, Matthew Kindig, Mehdi M Mirbagheri

**Affiliations:** 1 Deptartment of Physical Medicine and Rehabilitation, Northwestern University, Chicago, IL 60611, USA; 2 Sensory Motor Performance Program, Rehabilitation Institute of Chicago, Chicago, IL 60611, USA

**Keywords:** Locomotor training, Spinal cord injury, Modeling, Gait recovery, Gait impairment, Lokomat

## Abstract

**Background:**

Motor impairment is a major consequence of spinal cord injury (SCI). Earlier studies have shown that robotic gait orthosis (e.g., Lokomat) can improve an SCI individual’s walking capacity. However, little is known about the differential responses among different individuals with SCI. The present longitudinal study sought to characterize the distinct recovery patterns of gait impairment for SCI subjects receiving Lokomat training, and to identify significant predictors for these patterns.

**Methods:**

Forty SCI subjects with spastic hypertonia at their ankles were randomly allocated to either control or intervention groups. Subjects in the intervention group participated in twelve 1-hour Lokomat trainings over one month, while control subjects received no interventions. Walking capacity was evaluated in terms of walking speed, functional mobility, and endurance four times, i.e. baseline, 1, 2, and 4 weeks after training, using the 10-Meter-Walking, Timed-Up-and-Go, and 6-Minute-Walking tests. Growth Mixture Modeling, an analytical framework for stratifying subjects based on longitudinal changes, was used to classify subjects, based on their gait impairment recovery patterns, and to identify the effects of Lokomat training on these improvements.

**Results:**

Two recovery classes (low and high walking capacity) were identified for each clinical evaluation from both the control and intervention groups. Subjects with initial high walking capacity (i.e. shorter Timed-Up-and-Go time, higher 10-Meter-Walking speed and longer 6-Minute-Walking distance) displayed significant improvements in speed and functional mobility (0.033 m/s/week and–0.41 s/week respectively); however no significant change in endurance was observed. Subjects with low walking capacity exhibited no significant improvement. The membership in these two classes—and thus prediction of the subject’s gait improvement trajectory over time—could be determined by the subject’s maximum voluntary torque at the ankle under both plantar-and dorsi-flexion contractions determined prior to any training.

**Conclusion:**

Our findings demonstrate that subjects responded to Lokomat training non-uniformly, and should potentially be grouped based on their likely recovery patterns using objective criteria. Further, we found that the subject’s ankle torque can predict whether he/she would benefit most from Lokomat training prior to the therapy. These findings are clinically significant as they can help individualize therapeutic programs that maximize patient recovery while minimizing unnecessary efforts and costs.

## Introduction

A common focus during rehabilitation after spinal cord injury (SCI) is on promoting improvements in functional walking capacity. Fast and effective enhancement of gait function can enhance a patient's independence, life satisfaction, and subsequent reintegration into society as a fully-participating member. Body weight support treadmill training (BWSTT) has been considered a primary rehabilitative strategy for improving locomotion in patients with incomplete SCI [1,2]. BWSTT provides intensive, task-specific repetitive walking training, promotes supraspinal neuroplasticity involved in locomotion, and consequently helps patients to regain their walking skills [3-5]. While conventionally BWSTT is supplemented by manual assistance from therapists, robotic-assisted BWSTT has become widely used over the past decade to provide symmetric walking training to support lower extremity rehabilitation in patients with neurological movement impairment. The most widely-used automated locomotor training system is the Lokomat® (Hocoma AG, Volketswil, Switzerland) [6,7]. Recent studies have shown that patients can receive positive physical and psychological benefits from Lokomat training [1,2,8-17], such as improved walking capacity [1,8-10], improved metabolic performance [2,12], and increased activity in the cerebellum [15]. However, a recent review from Swinnen [18] has shown that the effectiveness of Lokomat training remains controversial, with some studies showing significant improvements in motor and walking performance after Lokomat trainings, while others showing minimal changes [18]. The authors and previous researchers have pointed out that difficulty arises when translating these research findings to the clinical setting because of the lack of a control group in those studies [19]. The authors also observe that the advantages of Lokomat training over other types of locomotor trainings have yet to be fully demonstrated.

In addition, the high variability in recovery pattern among patients should be explicitly considered in the analysis. Locomotor training, like any other intervention, is not expected to affect all patients equally; instead, different treatment responses are expected for different patient subgroups. Many research studies, however, oversimplify by assuming that all individuals are drawn from a single population with a similar recovery pattern; pre-training and post-training measures are then compared. This approach loses the heterogeneous response of patients to the therapy program as well as the longitudinal information describing the timeline of improvement—information that should be considered when evaluating the efficacy of a particular intervention. In fact, many studies involved with locomotor training have reported results with a standard deviation even larger than the sample mean, indicating that the high inter-individual variance within their sample population results in a less meaningful average value. Thus, it is necessary to address these deficits by first identifying distinct latent classes within each sample and then modeling the recovery patterns for each class separately. In addition, pre-vs. post-training comparisons usually have a lowered effect size and statistical power than longitudinal tracking studies, because the error term has a larger standard deviation than in other statistical models [20].

Recent statistical techniques such as Growth Mixture Modeling (GMM) allow researchers to effectively recognize this high inter-individual variance, and to model the growth pattern of a longitudinal recovery procress. The inclusion of additional time points between the pre-and post-training evaluations can help elucidate the training effect on gait impairment, and further can dramatically improve the statistical power in situations where increasing sample size is not feasible due to costs and patient availability [20].

In the present study, rather than performing a pre-vs. post-training comparison within one experimental treatment group, we examined in detail the growth trajectory of three aspects of gait impairment (speed, functional mobility, and endurance, as evaluated by typical clinical measures) for subjects in an intervention group receiving Lokomat training and those in a control group who received no intervention. Instead of assuming that all subjects are drawn from a single population, we objectively classified the subjects into distinct subgroups based on their growth trajectory of walking capacity, and then modeled the effect of Lokomat training for each subgroup. Finally, we evaluated whether baseline measurements of neuromuscular and clinical performance could serve as statistical predictors for these recovery patterns. In particular, we hypothesized that ankle strength—measured in terms of maximum voluntary dorsiflexion and plantarflexion torque—could predict the recovery trend for these clinical evaluations.

## Method

### Study participants

Forty incomplete SCI subjects (twenty-seven males, thirteen females) with spastic hypertonia at their lower extremities participated in this single-center, unblinded, and randomized study (Table 1). Each subject was assigned either to the control or intervention (Lokomat training) group according to a permuted block randomization design. All subjects were injured within their cervical or upper thoracic (superior to T10) vertebrae.

**Table 1:** Baseline characteristics of SCI patients (n = 40) in the Lokomat and control group (mean ± standard deviation), including age, post-injury time, ankle joint tested, gender ratio, walking index for spinal cord injury (WISCI II), plantarflexion MVC torque (Tp) and dorsiflexion MVC torque (Td)

**Group**	**Age (yrs)**	**Post-injury time (yrs)**	**Ankle joint tested (# of patients)**	**Gender ratio (Female/Male)**	**WISCI II**	**Plantarflexion MVC torque (Tp, Nm)**	**Dorsiflexion MVC torque (Td, Nm)**	**Paraplegia/Tetraplegia (# of patients)**
Lokomat (n = 20)	42.2 ± 12.6	8.9 ± 9.9	8 right/ 12 left	7/13	15 ± 4	25.5 ± 16.8	11.2 ± 6.2	6/14
Control (n = 20)	49.7 ± 7.0	7.5 ± 5.5	7 right/ 13 left	6/14	16 ± 4.5	28.7 ± 18.6	13.9 ± 8.2	6/14

Note: No significant difference was found for any baseline measure between groups (*P* = NS).

All subjects gave informed consent according to the policies of the Institutional Review Board of Northwestern University.

### Lokomat training

The Lokomat was used to train subjects in pre-programmed physiological gait pattern. The system uses a pair of robotic orthoses to move the subject’s legs over a synchronized treadmill while a harness system dynamically adjusts the amount of body-weight support. Gait parameters, such as the range of motion of the hip and knee joints, walking speed, body weight support, and guidance force, were adjusted in real-time by the physical therapist.

Each subject received a one-hour training session three times per week for four consecutive weeks; as it took 15–20 minutes to set up the subject, the gait training lasted up to 45 minutes per session. The goal of the Lokomat training was to increase the walking speed and decrease guidance force and body-weight support over the twelve training sessions based on the subject's improvement. In each session, the training speed was increased gradually from the minimum allowed by the machine to a peak magnitude (up to 3.4 km/hr) where the subject could maintain gait quality without feeling fatigued. The guidance force was then decreased from full assistance of the robot, until the subject was no longer able to maintain the set speed (low to 20% assistance) and the body-weight support, customized to the individual subject’s ability. The same therapist provided all trainings for a given subject.

### Outcome measures: walking capacity

Walking capacity was assessed during the training regimen using three standard clinical evaluations: 10-Meter-Walking Test (10MWT) [21], Timed-Up-and-Go (TUG) [22], and 6-Minute-Walking Test (6MWT) [23]. Subjects were evaluated four times for their overground walking capacity: at the baseline (i.e., prior to any Lokomat training), and after 1, 2, and 4 weeks of training. Subjects were permitted to use assistive devices and braces during the tests. Harness systems, parallel bars, and other support systems were not permitted. All tests for an individual subject were measured by the same examiner.

The 10MWT was used to evaluate walking speed over a short distance by measuring the time spent walking over a distance of 10 meters. The 10MWT has been rated as the test that provides the most valid measure of walking speed improvement [24].

The TUG was used to evaluate the functional recovery by measuring time taken by the subject to stand up from an armed chair, walk three meters, turn around, and sit back in the chair. It has been used to evaluate functional mobility and balance for various movement disorders [25-27].

The 6MWT test was used to assess walking endurance by measuring the distance walked in six minutes. It has been widely used to evaluate the response to therapeutic treatment for stroke and spinal cord injury, and is believed to provide a more accurate measure of daily activity than other walking tests [28].

These timed walking tests have been confirmed for their high concurrent and discriminant validity for SCI individuals [23,29], and are widely used as guidelines to both evaluate functional walking capacity and aid in the interpretation of clinical research results.

### Muscle strength: walking capacity predictor

The isometric torque resulting from the maximum voluntary contraction (MVC) at the ankle joint was measured after each subject finished all required clinical evaluations. Prior to any testing, the Modified Ashworth Score (MAS) was evaluated at each ankle joint. The ankle joint with the highest modified Ashworth scale and lowest maximum voluntary contraction (both conditions often occurred on the same side) was chosen for the MVC evaluation. Subjects were then seated in an adjustable chair with the ankle attached to a customized shoe mounted securely to a rigid plate. The trunk and thigh were strapped to the chair to minimize their contributions to ankle and foot movement. Subjects were then instructed to generate (MVC) in the dorsiflexion (Td) and plantarflexion (Tp) directions, using only their ankle joint, while the ankle joint was held fixed at 90° and the knee joint was held at 120°. During the experiment, the subject exerted a maximum force by pressing down (plantarflexion) or pulling up (dorsiflexion) against the stationary foot plate, which was in turn attached to a six-axis torque transducer (JR3 Inc., Woodland, CA). The subjects were instructed to practice exerting the MVC torque without using their knee and hip joints by minimizing the EMG activity in quadriceps and hamstring muscles. Electromyography (EMG) activity from the tibialis anterior, gastrocnemius, quadriceps and hamstring muscles was monitored using bi-polar surface electrodes (Ag-Ag/Cl type, Delsys, Boston MA); trials with substantial quadriceps and hamstring activity were repeated. Two trials were performed for each evaluation; the trial with the higher peak torque was chosen for the subsequent data analysis.

### Data analysis: recovery pattern identification and prediction

Growth Mixture Modeling (GMM) was used to subdivide participants into multiple latent classes based on the recovery patterns (i.e., the change over time) of their walking measures, and subsequently inspect gait improvement within each class. Logistical regression was then applied to identify potential predictors for subject class membership.

#### Recovery pattern identification using GMM

GMM is one type of longitudinal mixture model, and has been widely used to analyze data that exhibits heterogeneity in developmental pathways [30-37]. GMM assumes that a given sample of subjects can be divided into a finite number of groups, each of which is comprised of subjects with similar recovery patterns. That is, all subjects within a given class were assumed to exhibit similar changes over time. In effect, GMM is analogous to a clustering technique, e.g., *k*-means, but with consideration of multiple observations over time. The intervention effect was then be modeled within individual subgroups and compared with control or other treatment groups. A two-step GMM procedure was employed in this study. Step 1 classified the control and intervention groups separately, based on the measured growth trajectories of their clinical evaluations. A mixed-effects model (random coefficient regression) was then used to determine if the trajectories showed significant change over time. Step 2 involved a joint analysis to examine the training effect on walking capacity. For Class 1, the difference in the rates of change between the control and intervention groups was compared using the regression slopes; the same procedure was then used for Class 2 [37]. The quality of the resulting classification was evaluated by the posterior probabilities of subject class membership and by the Bayesian information criteria.

#### Prediction of recovery patterns at baseline using logistic regression analysis

The class membership of subjects in each treatment group was correlated with the latent variables (potential predictors) using logistic regression [38-40], in order to determine whether injury level, age, gender, walking index for spinal cord injury (WISCI II) and muscle strength (MVC torque at the ankle joint) evaluated at the baseline could predict clinical assessments of functional improvement over the subsequent four weeks. Such an analysis can help to discover potentially-causal relationships between the predictors and functional improvement.

The group allocation, i.e., control and Lokomat training, was concealed (blinded) to the statistician. The GMM analysis was performed in the R statistical package V 2.12 (open source software, http://www.r-project.org), while the logistic regression was performed using SAS v 9.0 (SAS Institute, Cary NC). Statistical tests with a *P <* 0.05 were considered significant.

## Results

### Recovery patterns of gait impairment measures

**Figure 1:**
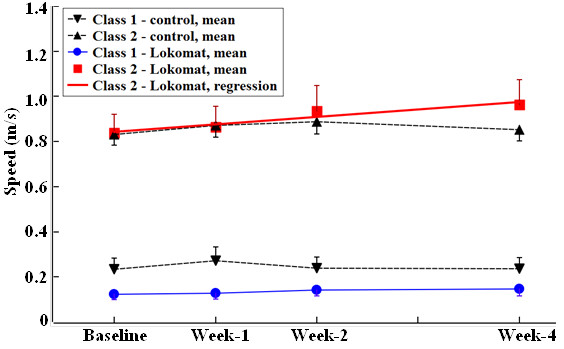
**10MWT speed as a function of training week.** Mean ± SE for each latent class shown. The linear regression line for Class 2 in the Lokomat group was obtained from GMM. No other changes in 10MWT speed with time were significant.

**Figure 2:**
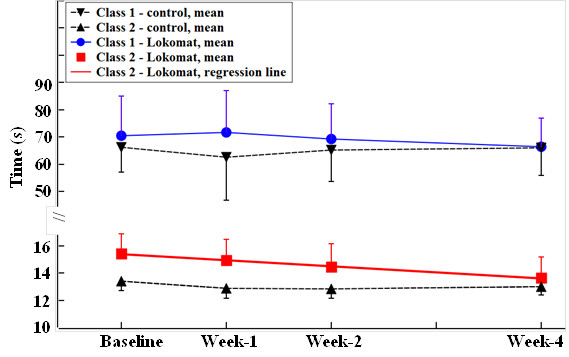
**TUG time as a function of training week.** Mean ± SE in each latent class shown. The linear regression line for Class 2 in Lokomat was obtained from GMM. Note that the ordinate scales are different between Class 1 and Class 2, due to their large difference in standard errors.

GMM was used to identify the growth trajectories for both control and intervention groups, and to identify the effect of training on these classes. Figures 1 and 2 show the mean trajectory for each class. Table 2 summarizes the statistical results from the GMM analysis.

**Table 2:** Illustration of classification and joint group analysis from GMM for control and intervention groups

	**Latent class**	**GMM analysis for control group**		**GMM analysis for intervention group**			**Joint group analysis of GMM**
		**# of subjects**	**Baseline measurement (mean ± SE)**	**# of subjects**	**Baseline measurement **	**Rate of change (P-value)**	**Difference in rate of change between two groups (P-value)**
10MWT	Class 1	8	0.233 ± 0.050 m/s	8	0.124 ± 0.024 m/s	0.007 m/s/week	0.009
	Class 2	12	0.829 ± 0.047 m/s	12	0.838 ± 0.083 m/s	0.033 m/s/week**	0.029*
TUG	Class 1	8	61.27 ± 9.16 s	6	70.49 ± 14.56 s	−3.35 s/week	−4.55
	Class 2	12	14.67 ± 0.81 s	12	15.04 ± 1.12 s	−0.41 s/week**	−0.32*
6MWT	Class 1	6	80.77 ± 18.33 m	6	42.27 ± 9.21 m	0.58 m/week	0.79
	Class 2	12	245.66 ± 21.02 m	12	279.40 ± 28.46 m	1.60 m/week	2.28

*Indicates p < 0.05, ** ndicates p < 0.01.

Note that in the control group two subjects who could not perform the 6MWT evaluation belonged to Class 1 for 10MWT and TUG, while two subjects in the intervention group who could not perform the TUG or 6MWT evaluations belonged to Class 1 for 10MWT.

#### Latent class identification

##### Control group

The growth trajectory for each of the three clinical measures was examined by GMM over the 4-week training period. Two distinct growth patterns were identified for each walking measure (Figures 1 and 2, Table 2). The latent classes that included subjects with longer 10MWT and TUG times and shorter 6MWT distances at baseline (i.e., the initial test) were defined as the low walking capacity classes (Class 1). Similarly, the latent classes for subjects with shorter 10MWT and TUG times and a longer 6MWT distance were defined as the high walking capacity classes (Class 2).

##### Intervention group

Similar to the control group, two classes of growth pattern were identified for each clinical evaluation, corresponding to low and high walking capacity at baseline (Figures 1 and 2, Table 2).

Subject class membership agreed across the different clinical evaluations for both groups. The average posterior probabilities of class membership were 0.99 for each walking test, suggesting low classification error.

#### Training effect identification

For the 10MWT test, the GMM analysis considering joint groups found a significant beneficial training effect for subjects with a high walking capacity (*P* = 0.02). In the Lokomat group, subjects in Class 2 increased their walking speed at a rate of 0.033 m/s/week (*P* = 0.01). Subjects in Class 1 did not show significant benefit from the Lokomat training (*P* = NS).

For TUG, the GMM analysis of joint groups found a beneficial training effect on subjects with high walking capacity (*P* = 0.05). Subjects in Class 2 of the Lokomat group decreased their time at a rate of 0.41 s/week (*P* = 0.02). Subjects in Class 1 did not show significant benefit from the Lokomat training (*P* = NS).

For 6MWT, the training effect was not found to be significant for subjects from either the high or low walking capacity groups, based on the GMM analysis (*P* = NS).

The change over time for each clinical measure was not significant for any latent class in the control group (*P* = NS).

### Prediction of gait impairment recovery patterns by mvc torque

**Figure 3:**
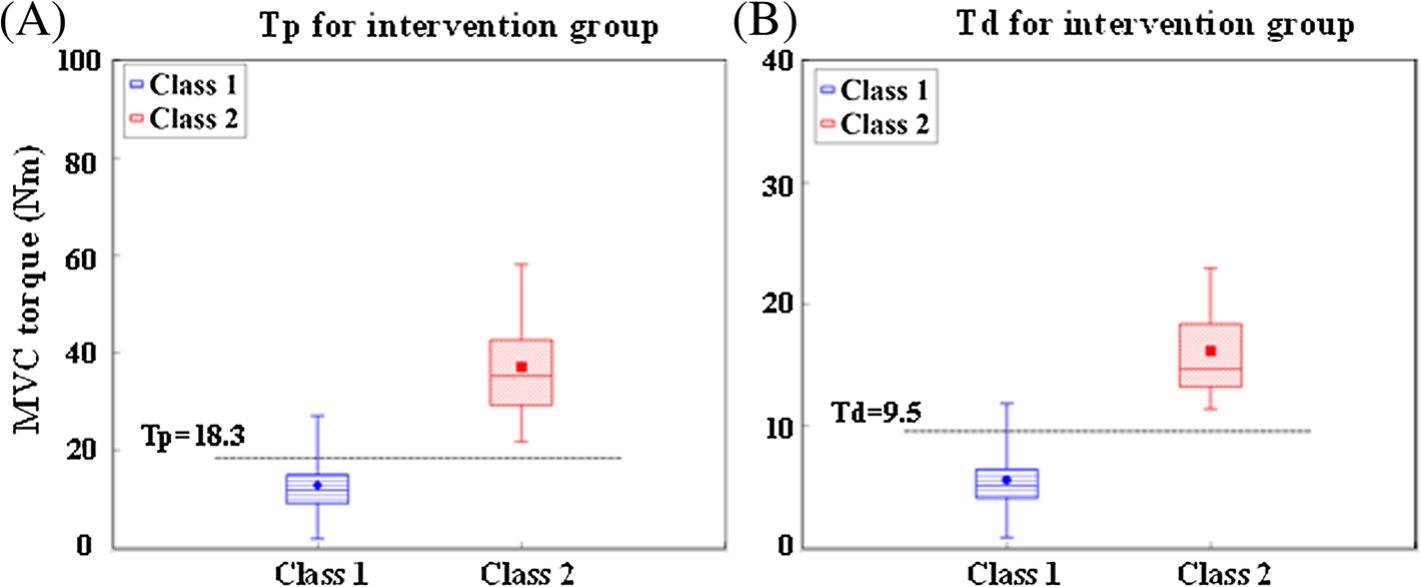
**Relationship between plantarflexion (Tp, panel A) torque and dorsiflexion (Td, panel B) MVC torque and subject class membership, for the 10MWT evaluation in the Lokomat group.** Note: in each boxplot, the bounds of the box denote the first and third quartiles, while the two whiskers denote the minimum and maximum values.

Figure 3 shows the relationship between baseline MVC torque and subject class membership for the 10MWT evaluation in the Lokomat group. Table 3 presents the results of the logistic regression to determine whether MVC torque is able to predict subject class membership for the Lokomat group.

**Table 3:** Logistic regression results used to assess whether Tp or Td could predict patient class membership in the Lokomat group

	**Logistic regression of class membership on Tp**			**Logistic regression of class membership on Td**		
	**Odds ratio [95% CI]**	**P-value**	**Baseline Tp (Nm)**	**Odds ratio [95% CI]**	**P-value**	**Baseline Td (Nm)**
**Class 2 in 10MWT**	1.18 [1.05, 1.41]	P < 0.01	>18.3	2.20 [1.26, 8.22]	P < 0.01	>9.5
**Class 2 in TUG**	1.16 [1.03, 1.39]	P < 0.01	>17.5	2.18 [1.23, 8.22]	P < 0.01	>9.5
**Class 2 in 6MWT**	1.18 [1.04, 1.45]	P < 0.01	>16.1	2.10 [1.28, ∞]	P < 0.01	>8.9

The relation between baseline torque (Tp or Td) and patient class membership was measured by the odds ratio with Class 1 of each clinical measure as the reference group.

As the plantarflexion MVC torque (Tp) had overlapping ranges for the two latent classes for each clinical evaluation, logistic regression was used to inspect the effects of Tp on the subject class membership, using Class 1 as the reference. For the 10MWT evaluation, subjects with Tp > 18.3 Nm were more likely to be members of Class 2 than subjects with Tp ≤ 18.3 Nm. For each unit increase in Tp, the odds that a subject belonged to Class 2 increased by 18% (*P* < 0.01). For the TUG test, it was shown that subjects with Tp > 17.5 Nm had a higher likelihood of membership in Class 2 (*P <* 0.01). For 6MWT, subjects with Tp > 16.1 Nm were more likely to belong to Class 2 than subjects with Tp ≤ 16.1 (*P* < 0.01).

A similar logistic regression was performed for the dorsiflexion MVC torque (Td). It was shown that subjects with Td > 9.5 Nm had a higher likelihood of Class 2 membership than subjects with Td ≤ 9.5 Nm for both the 10MWT and TUG evaluations (*P* < 0.01). For the 6MWT, subjects with Td > 8.9 Nm were more likely to belong to Class 2 than subjects with Td ≤ 8.9 Nm (*P* < 0.01).

**Figure 4:**
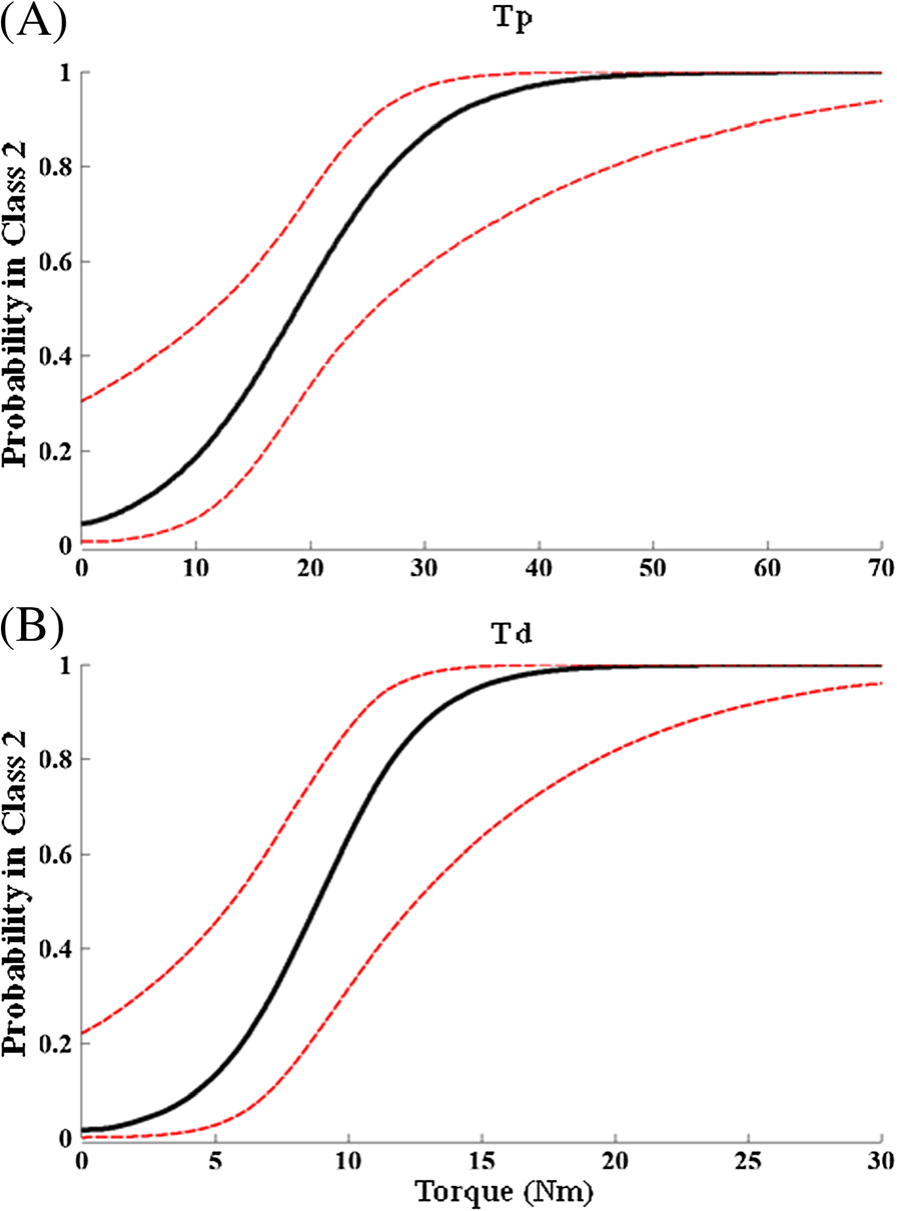
**Logistic regression results to determine ability of Tp (panel A) and Td (panel B) to predict class membership.** Regression indicates whether MVC torque predicts whether a subject is in the high walking capacity class (i.e., Class 2) for pooled subjects from both groups. The predicted probability curve (solid line) and 95% confidence limits (dashed line) are shown.

Figure 4 shows the predicted probability of a subject to belong to the high walking capacity class (Class 2), based on their measured Tp or Td, assuming pooled subjects of both the control and intervention groups. It further confirmed that Tp and Td were significant predictors for walking-capacity classification (*P* < 0.01).

Other possible factors, including injury level, Modified Ashworth Scale score, gender, age and post-injury time were found not to have any significant effect on the class membership for any clinical evaluation (*P* = NS for all).

## Discussion

The longitudinal growth pattern of gait impairment for two groups of SCI subjects—control and intervention—over four weeks of ongoing Lokomat training, was investigated and analyzed with the GMM. Two classes of growth trajectory in each clinical evaluation were identified for both the control and intervention groups, representing subgroups with low and high walking capacity. The two latent classes responded to the Lokomat training differently; subjects with high walking capacity presented significant and consistent improvements in walking speed and balance with time, while subjects with low walking capacity exhibited no such significant improvement. Interestingly, the results indicated that the baseline (i.e. pre-training) measures of MVC torque (Td and Tp) could predict the differential treatment response, i.e., subjects with high Tp and Td were more likely to have both high walking capacity and receive significant benefit from Lokomat training. These findings can help clinicians to identify the subgroups of individuals with incomplete SCI in terms of their current and potential walking capacity and to estimate the differential treatment effects of the Lokomat intervention on their recovery progress.

### The effect of lokomat training on walking improvement

The twelve sessions of Lokomat training for SCI subjects improved their functional ambulation in a selective manner. Subjects with low walking capacity did not show significant improvements. By contrast, subjects with a high walking capacity at baseline presented a consistent linear trend in time for both speed and functional balance over the 4-week training period. Our average improvement in 10MWT speed (0.132 m/s) indicates that the 12-session Lokomat training program can improve the walking speed for SCI subjects with high walking capacity; this improvement exceeds the threshold established by Lam [41] (viz. 0.13 m/s) for significant clinical change in 10MWT. Our results agree with previous reports that Lokomat training improves the overground walking capacity [1,9,15,42], and that subjects with less control impairment achieve greater improvement [19].

While the 10MWT, TUG and 6MWT evaluate different aspects of gait impairment, they have been shown to be highly-correlated in the walking validity test [23,29,41,43,44]. Despite this, surprisingly we observed no significant change in the 6MWT, whereas the 10MWT and TUG improved significantly. Such a discrepancy was also reported in a similar training for the stroke population [45]. By contrast, other studies reported improvements in endurance for SCI subjects who were provided with two months or more of Lokomat training as well as supplementary pharmaceutical intervention [1,10]. Thus, lack of significant improvement in 6MWT in our study could be due to the lower number of training sessions in our study than others. In particular, it is likely that the 4-weeks of training was insufficient for the individuals in the low-capacity class, as 4-weeks may be too little to effect the necessary neuroplasticity to improve walking function. Further, our walking endurance test was performed at a self-selected speed that the subject felt was most comfortable. Therefore, trial-to-trial variations during the clinical evaluations might be much higher than the improvements received from the training, i.e., an individual subject did not necessarily perform the clinical evaluations at his/her maximum effort level, complicating the detection of the training effects.

### Need for classification of recovery patterns

A few other studies in the literature have classified their participants prior to other analyses, based on *a priori* or empirical considerations. A walking validity test by van Hedel et. al. grouped subjects into low and high capacity based on their walking index score prior to performing the correlation analysis [23]. Correlation between walking tests and WISCI II score was found to be significant for subgroup with high walking function (WISCI II > 10), while it was little for subgroup with WISCI II ≤ 10. A study by Field-Fote et. al. also classified subjects into two subgroups by their walking speeds [9]. They found that subjects with slower initial walking speed achieved higher improvement in walking speed than those with faster initial walking speeds, in contrast with our finding. The discrepancy could be due to the fact that they used a limited number of subjects who were randomly assigned to 4 different body-weight-support assisted-stepping groups, including treadmill training with manual assistance, robotic assistance, stimulation, and overground training with stimulation. Each slower- and faster- initial walking group included a combination of subjects who received different types of training with different mechanisms of actions, making the interpretation of the effects of Lokomat alone difficult.

By contrast, the GMM analysis in this study classified subjects with the same training in terms of their growth trajectories using a rigorous statistical technique, which provides objective evidence for the validity of classifying subjects into subgroups prior to other statistical analyses.

Further, it can be expected that the recovery manner of SCI subjects during neurorehabilitation might present more complex growth patterns than those found in the current study, e.g., different numbers of latent classes for different clinical tests, and inconsistencies in the class membership between the various clinical evaluations. Classification with objective criteria, such as that provided by GMM, can provide a robust and repeatable analytical framework to reveal distinct subgroups which exhibit substantial differences between subgroups but maintain high within-group similarities.

### Prediction of effect of lokomat training on improving walking capacity

To determine the extent to which the recovery pattern of walking capacity can be predicted based on baseline measures, the ability of MVC torque (Td and Tp) to determine subject classification was assessed. Our results showed that subjects with a larger Tp or Td had a higher likelihood of membership in the high walking capacity group for each clinical test, and thus to receive significant training benefit. Such findings indicate that this quantitative measurement of ankle MVC dorsiflexion and plantarflexion torque can potentially be a fast and reliable clinical assessment that can be used to identify which subjects are most likely to make significant progress during a one-month Lokomat training regimen prior to the start of training. The ability of other kinetic and kinematic variables to predict gait recovery, such as knee and hip strength, as well as range of motion of lower limb joints, will be investigated in future studies.

Although clinical assessments of gait impairment, such as the 10MWT, TUG, and 6MWT assessments, have been well-accepted by physicians, their applications are also limited by the requirement of necessary time and space to perform such tests, inter-subject differences due to the choice of route for the 6MWT assessment, and the motivation for subjects to perform the tests [28]. By contrast, our study implies that ankle MVC provides an objective measurement that can be part of a routine office visit with a physician.

Our study also can lend insight into the role of muscle strength on gait impairment. In particular, we expect that weakness of the anterior tibialis (TA) and the gastrocnemius (GS) are likely related to gait impairment. This is expected due to the role played by the neuromuscular properties of the spastic ankle during ambulation. For example, walking capacity after SCI is commonly impaired by equinus foot, partially due to spasticity and weakness of the ankle dorsiflexors. Spasticity of ankle plantarflexors has reciprocal inhibitory effects on TA activity, and additionally causes spastic hyperactivity of GS and hypoactivity of TA. Both of these can reduce the voluntary contraction capacity of these muscles and contribute to equinus foot and walking impairment. It has been shown that, in addition to improving walking capacity, Lokomat training can significantly reduce the neuromuscular abnormalities associated with spasticity and may result in increased ankle MVC torque [46]. Such an explanation is further supported by the finding that the improvements in muscle strength for SCI subjects with spastic hypertonia at the ankle joint are mainly due to reductions in reflex stiffness [47,48]. Our results extend those findings by showing that MVC torque measured at baseline can be a significant predictor for the recovery pattern of SCI gait impairment.

### Study limitations

It was not feasible to blind the researchers who performed the tests to the group allocation since they needed to stay with the subjects during the Lokomat trainings and experimental protocols. All tests and trainings for each individual subject were performed by the same researcher; however, since this longitudinal study occurred over a span of multiple years, it was not feasible for one researcher to test all subjects. The effects of these limitations on the results are unclear.

### Directions for future research

Although several studies have compared the therapeutic effects of Lokomat to other physical interventions, it is our interest to use the advanced techniques that we used in this study to compare the therapeutic effects of this relatively new physical intervention to more well-established pharmacological interventions that are developed to improve function and reduce clinical impairments. Determining whether the Lokomat training is equally or more effective than pharmacological treatments is clinically significant, as some patients are able to tolerate physical interventions but not medications, while for others the opposite case is true. Thus, it is important to quantify outcomes for different types of interventions in order to provide optimal treatments. Furthermore, since the mechanisms and actions of these interventions are different, applying a combination of these interventions may provide benefits superior to the individual treatments.

## Conclusions

Overall, our study indicates that Lokomat training can be an effective physical intervention for SCI subjects with high walking capacity, but not for those with low walking capacity. Further, our findings indicate that the MVC torque measured prior to training may be an objective and reliable tool to predict which subject groups can benefit the most from Lokomat training, prior to being assigned to that therapy. Such an approach can allow clinicians to prescribe a therapeutic and rehabilitation plan that best optimizes an individual’s recovery outcomes while minimizing unnecessary effort and costs.

## Competing interests

The authors declare that they have no competing interests.

## Authors’ contributions

XN and DV conducted the experimental procedure and conducted most of the data processing and statistical analysis. XN primarily drafted the manuscript, with substantial editorial assistance from MMM and MK. MMM conceived and supervised the project, and oversaw all experimental and analytical efforts. All authors read and approved the final manuscript.
